# An Examination of Diet for the Maintenance of Remission in Inflammatory Bowel Disease

**DOI:** 10.3390/nu9030259

**Published:** 2017-03-10

**Authors:** Natasha Haskey, Deanna L. Gibson

**Affiliations:** Department of Biology, The Irving K. Barber School of Arts and Sciences, University of British Columbia, Room, ASC 368, 3187 University Way, Okanagan campus, Kelowna, BC V1V 1V7, Canada; natasha.haskey@gmail.com

**Keywords:** diet, diet therapy, nutrition, nutrition therapy, inflammatory bowel disease, ulcerative colitis, Crohn’s disease

## Abstract

Diet has been speculated to be a factor in the pathogenesis of inflammatory bowel disease and may be an important factor in managing disease symptoms. Patients manipulate their diet in attempt to control symptoms, often leading to the adoption of inappropriately restrictive diets, which places them at risk for nutritional complications. Health professionals struggle to provide evidence-based nutrition guidance to patients due to an overall lack of uniformity or clarity amongst research studies. Well-designed diet studies are urgently needed to create an enhanced understanding of the role diet plays in the management of inflammatory bowel disease. The aim of this review is to summarize the current data available on dietary management of inflammatory bowel disease and to demonstrate that dietary modulation may be an important consideration in managing disease. By addressing the relevance of diet in inflammatory bowel disease, health professionals are able to better support patients and collaborate with dietitians to improve nutrition therapy.

## 1. Introduction

Inflammatory Bowel Disease (IBD) is a chronic inflammatory condition that includes ulcerative colitis (UC) and Crohn’s disease (CD). CD is a transmural inflammatory disease of the gastrointestinal mucosa that can affect any component of the gastrointestinal tract including the small and/or large intestine, the mouth, esophagus, stomach and anus. UC is non-transmural and primarily involves only the colonic mucosa. Patients with IBD experience periods of relapse (active disease) and clinical remission [1]. Clinical symptoms of IBD are diarrhea and/or constipation, passage of blood or mucus in the stool and abdominal cramping. In addition, CD patients may also experience bowel obstruction, strictures and abscesses. 

Medications are the first-line of therapy in the management of IBD. Surgery is generally reserved for those patients who are intolerant to medications or have refractory disease. The main classes of drugs used in the treatment of IBD include 5-aminosalicylates (5-ASA) (e.g., sulphasalazine, and mesalamine), corticosteroids (e.g., hydrocortisone, prednisone, and prednisolone), immunomodulators (e.g., azathioprine, 6-mercaptopurine, methotrexate and cyclosporine) and biologics (e.g., infliximab, adalimumab, and vedolizumab). Although many patients have a good response to pharmacotherapy, medications can have severe adverse side effects. Drug hypersensitivity, nephrotoxicity, fever, rash, lymphadenopathy, hepatitis, pancreatitis, diarrhea exacerbation, nausea, vomiting, abdominal pain, myalgia and an increased risk for lymphoma with the use of immunomodulators are some of the reported side effects. In addition, medication adherence is a concern, as a recent study reported that approximately one quarter of patients with IBD attending at a tertiary care practice did not use the prescribed IBD-specific medications [2]. It is clear the adoption of novel strategies and preventative medicine is crucial to the well-being of patients with IBD to avoid complications and side-effects related to both the disease, as well as the treatments.

Exclusive enteral nutrition is a nutritional therapy where 100% of a patient’s nutritional requirements are met via a liquid nutrition formula and administered orally or through a feeding tube. Exclusive enteral nutrition is provided for 6–8 weeks and then an oral diet is gradually reintroduced [3]. In children with active CD, exclusive enteral nutrition can be used in place of corticosteroid therapy and has been shown to improve time to relapse [4]. This approach has also been shown to positively impact inflammatory changes, mucosal healing, enhance growth and overall nutritional status in children [4]. Outside of Japan, exclusive enteral nutrition is not routinely used as first-line therapy in adults. There is some evidence to support the use of exclusive enteral nutrition as a treatment modality in a select group of adult patients with CD, specifically those with a new diagnosis of CD, those with ileal involvement and those that are motivated to adhere to the exclusive enteral nutrition regimen [5]. 

IBD is associated with nutritional complications during both relapse and clinical remission. Some of the nutritional complications include reduced dietary intake, nutrient malabsorption, macro- or micronutrient deficiencies, weight loss and osteoporosis [6,7,8,9,10]. IBD patients are concerned about food and diet [11,12] and use various dietary strategies in attempt to control or minimize gastrointestinal distress [13], as well as improve overall health. Up to 71% of patients with IBD believe diet affects their disease symptoms, with 90% of CD patients and 71% of UC patients employing elimination diets while in remission [14,15]. Dietary modification can be of concern if patients drastically reduce or completely avoid nutritionally important foods/food groups, as this may place them at increased risk for developing nutritional deficiencies [16], as well as poorer quality of life. Up to 77.1% of patients with IBD report avoidance of particular foods [14]. A case-control study found significantly lower mean daily intakes of carbohydrates, monounsaturated fat, fiber, calcium, and vitamins C, D, E, and K compared to controls, as a result of the exclusion of dairy products, vegetables and fruit [17].

**Figure 1:**
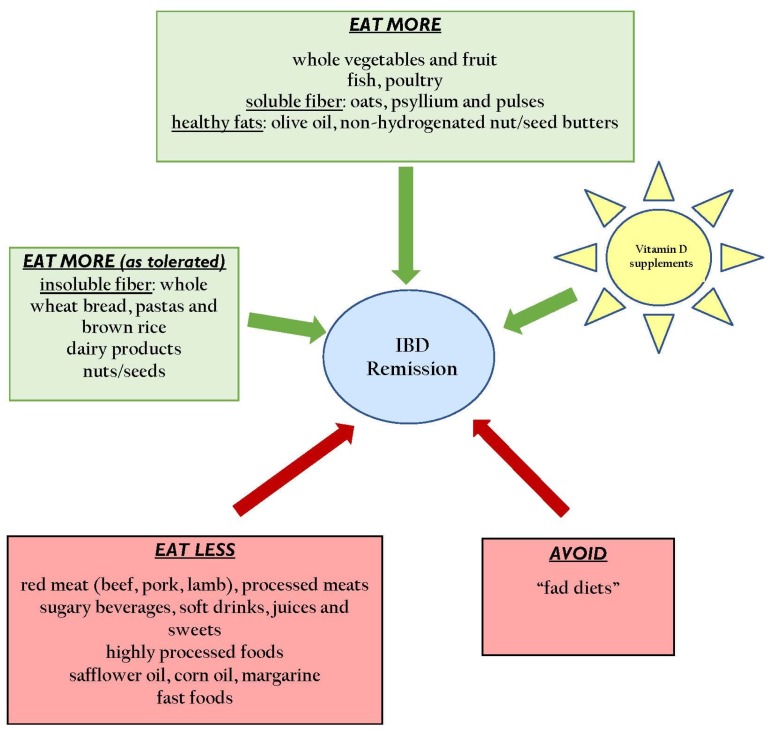
Summary of practical dietary recommendations for maintenance of remission in IBD.

There is a plethora of information on the internet that assert that certain diets improve or exacerbate symptoms, yet few rigorous nutrition studies have examined specific dietary factor(s) as being detrimental or protective against UC or CD. Implementation of diet strategies and approaches into clinical practice are slow and limited. Common barriers to implementation into practice include issues relating to knowledge management, such as access to research evidence sources, time to review evidence-based sources and a lack of skills to appraise and understand research evidence [18]. In addition to knowledge management, financial barriers, inappropriate skill mix and problems working with and across professional disciplines contribute to the lag in knowledge translation [18]. This review attempts to consolidate the existing evidence on diet/pharmoconutrition and maintenance of remission in IBD, including studies that evaluate the association between pre-illness intake of nutrients and food groups and the risk of a subsequent diagnosis of IBD, diet intervention studies in maintaining remission in IBD, as well as existing guidelines [19,20,21], in light of the most current limitations in evidence and practice. Evidence-based dietary recommendations for IBD patients are summarized in Table 1 and Table 2 and Figure 1. 

## 2. Literature Search

A systematic literature search was conducted using PubMed, EMBASE and Medline from 1966 to October 2016 using the medical subject heading (MeSH) terms “inflammatory bowel disease”, “Crohn’s disease”, “ulcerative colitis”, “nutrition”, “nutrition therapy”, “diet” and “diet therapy”. Searches containing relevant synonyms and combinations of the above terms were also utilized. We reviewed intervention studies, systematic reviews, as well as relevant review articles that contained practice guidelines with a focus on adults. Studies covering active IBD, enteral/parenteral nutrition and IBD with colectomy/ostomy were excluded from the review.

## 3. Diet in the Etiology of IBD

IBD has traditionally been thought of as a disease of the Western hemisphere, however there is an increasing incidence in Japan, Hong Kong, Korea and Eastern Europe [62,63]. Although still rarer, an increasing incidence of IBD is also being identified in South Africa, South America and Saudi Arabia [64,65,66]. The dramatic rise in incidence of IBD, particularly in South Asia, India and Japan, where traditionally there was a low incidence, suggests that environmental factors, such as the Western diet pattern, play an important role in disease pathogenesis [67,68,69]. This hypothesis is further confirmed by the increasing incidence of the disease in immigrants to the Western hemisphere. Migration from a country with a history of low-incidence to a country of a higher incidence increases the risk of developing IBD, particularly in the first generation children [70]. Diet composition has long been suspected to contribute to IBD. Thus, dietary patterns and nutrients are important environmental factors to consider in the etiology of IBD [22,71].

### 3.1. The Western Diet Pattern

The diet of today is considerably different from the traditional diet of previous generations, when the prevalence of IBD was considerably lower. The Western diet pattern is dominated by increased consumption of refined sugar, omega-6 polyunsaturated fats and fast food, combined with a diet deficient in fruit, vegetables, and fiber [72]. Much of today’s food supply has been processed, modified, stored and transported great distances, in contrast to the traditional diet, where food that was produced locally was consumed shortly after harvest. This shift to the Western diet pattern is hypothesized to increase pro-inflammatory cytokines, modulate intestinal permeability, and alter the intestinal microbiota promoting a low-grade chronic inflammation in the gut [73]. A diet that contains pro-inflammatory foods is an important risk factor in the development of UC. A case-control study completed in Iran with newly diagnosed UC patients (*n* = 62 UC patients, 124 controls) found that subjects that had a higher dietary inflammatory index (pro-inflammatory diet) had an increased risk of developing UC (Odds Ratio (OR): 1.55, 95% Confidence Interval (CI): 1.04–2.32) [23]. The authors concluded that encouraging intake of more anti-inflammatory dietary factors, such as plant-based foods rich in fiber and phytochemicals, and reducing intake of pro-inflammatory factors, such as fried or processed foods rich in trans-fatty acids, could be a potential strategy for reducing risk of UC. This was one of the first studies that has examined dietary inflammatory index as an outcome for developing UC. Several large scale studies have attempted to elucidate the dietary components that are associated with IBD risk [22,24,26,27]. Overall, this suggests that the Western diet pattern is a risk factor for IBD.

### 3.2. Carbohydrate Intake as a Risk Factor for IBD

A systematic review (*n* = 19 studies with 2609 IBD subjects) reported a negative association between dietary fiber (OR 0.12, 95% CI: 0.04–0.37) and fruit intake (OR: 0.2, 95% CI: 0.1–0.9) and CD risk [22]. Soluble fiber from fruit may have a protective effect on CD [24]. High vegetable intake may be associated with decreased risk of UC (OR range 0.32–0.75) [22]. The European Investigation into Cancer and Nutrition study (*n* = 366,351 with 256 incident cases of UC and 117 of CD, and four matched controls per case) reported that an increased consumption of sugar and soft drinks with low vegetable intake was positively associated with UC risk (OR 1.31, 95% CI: 0.85–2.02; *p* = 0.05) [25]. Increased consumption of sweets is positively associated with CD (OR: 2.83, 95% CI: 1.38–5.83) and UC (OR: 2.86, 95% CI: 1.24–6.57) [30]. Overall, this suggests that while refined and processed carbohydrates and intake of sweetened beverages are risk factors for IBD, complex carbohydrates including fruit, vegetables and fiber should be included in the diet to manage IBD.

### 3.3. Protein Intake as a Risk Factor for IBD

A large prospective cohort study (*n* = 67,581) completed over a 10.5-year period found that high protein intake, specifically animal protein (meat, not dairy products) was positively associated with an increased risk of IBD [31]. A systematic review (*n* = 2609 IBD patients; 19 studies) reported an association with high total protein intake with the development of UC (OR range 0.2–3.7) and CD (OR range 0.45–3.34) [22]. High protein intake was associated with a 3.3-fold increased risk of IBD, suggesting a diet high in animal protein is a major risk factor for the development of IBD.

### 3.4. Dairy Intake as Risk Factor for IBD

The European Investigation into Cancer and Nutrition study found that individuals that consumed milk had significantly reduced odds of developing CD (OR: 0.30, 95% CI: 0.13–0.65), suggesting a protective effect with dairy product consumption [28]. Individual dairy products consisted of milk, yogurt, and cheese with varying fat content (e.g., full fat, skimmed, semi-skimmed, and unspecified). This is supported by a case-control study in children (*n* = 130 CD patients and *n* = 202 controls) that demonstrated that consumption of dairy products was not associated with CD (OR: 0.86, 95% CI: 0.42–1.76, *p* = 0.65) [29]. Overall, the consumption of dairy products is not a risk factor for IBD.

### 3.5. Dietary Fat Intake as a Risk Factor for IBD

There have been conflicting data on the association between dietary fat intake and the development of IBD, as many of the studies are retrospective and use small sample sizes. However, a very large, long-term, prospective study (*n* = 170,805) completed over 26 years did not observe a significant association with increased risk of developing CD or UC with total dietary fat intake, saturated fatty acids (SFA) and monounsaturated fatty acids (MUFA) [26], which has been well supported by other research studies [74,75,76]. A growing body of scientific evidence indicates that the Mediterranean diet pattern has been associated with significant improvements in health status [77,78] and decreases in inflammatory markers in humans [79]. The protective effect is hypothesized to be derived from the balance in fats, which includes incorporating MUFA, SFA and fish intake [80]. While a few studies do show that MUFAs are beneficial during colitis, studies on the effects of SFA and PUFAs on gut health are controversial.

Dietary *n*-6 PUFA, in particular linoleic acid, have been implicated in the etiology of IBD. Dietary *n*-6 PUFAs are essential fatty acids present in high amounts in red meat, cooking oils (safflower and corn oil) and margarines. A prospective cohort study (*n* = 203,193) conducted over four years found that intake of linoleic acid was associated with an increased risk of UC (OR: 2.49, 95% CI: 1.23 to 5.07, *p* = 0.01) [27]. Further analysis of the European Investigation into Cancer and Nutrition study (*n* = 260,686) over five years found an increased risk of UC with a higher total PUFA intake (trend across quartiles OR = 1.19 (95% CI: 0.99–1.43) *p* = 0.07) [74], which was also supported by a systematic review (*n* = 2609 patients with IBD) that examined pre-illness intake of nutrients and subsequent development of UC [22]. A case-control study in CD found that increased total PUFA consumption was positively associated with CD risk (OR: 2.31, 95% CI: 1.12–4.79) [30].

The Nurses’ Health Study cohorts (*n* = 170,805 women with 269 incident cases of CD and 338 incident cases of UC) reported high, long-term intake of trans-unsaturated fatty acids was associated with a trend towards an increased incidence of UC (HR 1.34, 95% CI: 0.94–1.92) but not CD [26]. An increased relative risk of developing IBD has also been associated with frequent intake of fast foods (fast foods are high in trans-unsaturated fatty acids) [81,82]. The relative risk associated with the consumption of fast foods at least two times a week was estimated at 3.4 (95% CI: 1.3–9.3) for CD and 3.9 (95% CI: 1.4–10.6) for UC [82]. Frequent fast food intake, defined as more than once a week, was significantly associated with a risk of UC (43%, OR: 5.78, 95% CI: 2.38–14.03) and CD (27%, OR: 2.84, 95% CI: 1.21–6.64) [81].

It has been speculated that the intake of long-chain *n*-3 PUFAs (docosapentaenoic acid, eicosapentaenoic acid, docosahexaenoic acid), known as omega-3s, may be of benefit to patients with IBD. The beneficial effects are believe to be derived from the anti-inflammatory properties of *n*-3 PUFAs; however, clinical and experimental studies have shown conflicting results [83]. Meta-analyses have failed to show benefit with supplementation with fish oils in the maintenance of remission in CD and UC [84,85,86]. Dietary intake of *n*-3 PUFAs were inversely associated with risk of UC, whereas no association has been found with CD [26]. The European Investigation into Cancer and Nutrition study (*n* = 203,193) found a negative association with the development of UC with increasing dietary intake of the *n*-3 PUFA, specifically docosahexaenoic acid (OR: 0.23, 95% CI: 0.06 to 0.97) [27], and is supported by the European Investigation into Cancer and Nutrition -Norfolk study (*n* = 26,639) (OR: 0.43, 95% CI: 0.22–0.86) [57]. Two case-control studies in CD report that a diet with regular consumption of fish had a protective effect on the development of CD (OR 0.52, 95% CI: 0.33–0.80, *p* = 0.003) and (OR 0.46, 95% CI: 0.20–1.06, *p* = 0.02) [29,69]. 

The total ratio of *n*-3 PUFA: *n*-6 PUFA found in the diet has been hypothesized to be an important consideration. One prospective cohort [87] and one case-control study [29] report that a high *n*-3PUFA: *n*-6 PUFA ratio in the diet is inversely associated with the risk of IBD. In support of this explanation, a dietary intervention trial that focused on increasing the *n*-3 PUFA: *n*-6 PUFA ratio was found to be effective in maintaining disease remission in patients with both UC and CD, through increasing *n*-3 PUFA intake [88]. Overall, it does not appear that full fat diets should be avoided, however fat including diets rich in olive oil, dairy products and fish but not fish oil pills should be consumed while avoiding large intakes of vegetable oils rich in *n*-6 PUFA.

In summary, several epidemiological studies provide compelling evidence for the role of food in IBD pathogenesis. Furthermore, the rise in incidence of IBD in countries that previously have had a very low incidence suggests that industrialization and adoption of the westernized diet may be a risk factor in the development of IBD. Reduced consumption of fruits and possibly vegetables, resulting in a reduced overall intake of fiber, with high intake of meats, fast foods and trans-fatty acids appears to be associated with an overall increase in the risk of developing IBD [71].

## 4. Diet Interventions and IBD

Diet interventions have been studied in IBD in attempt to manage active disease or to maintain remission. A number have been shown to be efficacious, however, the precise components that are important for each diet are not clearly delineated or often contradict one another. With no gold standard, nutrition guidance provided at this time by health professionals is based on the “best available evidence”.

### 4.1. Low Residue Diet

A low-residue diet is often recommended for the management of an acute flare of IBD, especially in patients that have intestinal strictures or narrowing. Although the low-residue diet is prescribed for short-term use, in clinical practice, patients often follow the diet long-term. The primary purpose of a low residue diet is to reduce the frequency and volume of stools and reduce the risk for intestinal obstruction [89]. In the literature, there have been discrepancies as to the actual composition of low-fiber and/or low-residue diets. A low-residue diet requires the elimination of whole grains, legumes and all fruits and vegetables (except for bananas and skinless potatoes), dairy and fibrous meats [89]. This is not the same as a low-fiber diet which excludes only insoluble fiber. A prospective study in subjects with active CD (*n* = 70) that compared a low-residue diet to an unrestricted diet found no differences in outcome including symptoms, need for hospitalization, need for surgery, new complications, nutritional status, or postoperative recurrence [42]. Due to lack of evidence, there appears to be no reason to restrict residue from the diet, however anecdotally CD patients with obstructive symptoms and strictures report improvement in symptoms when following a diet reduced in fiber (total daily fiber intake < 10 g) [89,90].

Research is still in its infancy, however there is growing evidence for an association between IBD and an alteration in the gut microbiota. The Westernized diet, characterized by increased consumption of PUFA, animal protein, and sugar as well as decreased consumption of fiber has been implicated as factor contributing to dysbiosis [91,92,93]. A small pilot study in CD (*n* = 6), reported a marked decrease in microbial diversity with a low-residue diet [94]. This is concerning considering that low diversity of the microbiota has been linked to a variety of chronic diseases [95]. Furthermore, improvements in inflammatory markers have not been demonstrated with a low-residue diet in CD [96]. More research is required; however, a low-residue diet could potentially have negative consequences on IBD, therefore prolonged avoidance of fiber is discouraged [90].

### 4.2. Semi-Vegetarian Diet

A prospective trial conducted in Japan in hospitalized subjects with CD (*n* = 22) examined the effect of a semi-vegetarian diet on maintaining remission [43]. The diet was lacto-ovo vegetarian, in which eggs and milk were allowed with small portions of meat offered once every two weeks and fish weekly. Remission rate achieved with the semi-vegetarian diet was 100% after one year and 92% after two years. A semi-vegetarian diet showed significant prevention of relapse compared to that of individuals following an omnivorous diet (*p* = 0.003 log rank test). Based on these observations, the semi-vegetarian diet may be a highly effective way to maintain remission CD. While this study is promising, large, randomized control studies are required to validate the efficacy of this type of diet for IBD patients.

### 4.3. FODMAPs Diet

The low Fermentable Oligosaccharide, Disaccharide, Monosaccharide, and Polyol diet (FODMAPs) consists of eliminating foods high in fermentable but poorly absorbed carbohydrates and polyols for six to eight weeks [97]. FODMAPs comprise fructose, lactose, fructo- and galacto-oligosaccharides (fructans and galactans), and polyols (sorbitol, mannitol, xylitol and maltitol) [97].

Common food sources of foods containing FODMAPS are as follows: (1) fructans: onion, garlic and wheat; (2) fructose: fruits and fruit products, honey, and foods with added high-fructose sweeteners; (3) lactose: mainly dairy products; (4) oligosaccharides: legumes, nuts, seeds, some grains; and (5) polyols: fruits and vegetables, and sugar-free products. After symptom resolution, patients are guided by a dietitian on how to gradually reintroduce foods high in fermentable carbohydrates to determine individual tolerance to specific FODMAPs.

Fair evidence supports the effectiveness of a low-FODMAP diet for the symptom management of irritable bowel syndrome (IBS), especially a reduction in abdominal bloating, pain, and diarrhea [98,99]. IBS-like symptoms are common in IBD and have been reported in 57% of patients with CD, and 33% of patients with UC [100], therefore a low-FODMAP diet has been proposed for the management of patients with IBD with IBS-overlay. There have been three retrospective studies evaluating the low-FODMAP diet in IBD [32,33,34]. A retrospective study of 72 IBD patients who received dietary intervention focusing on low-FODMAP diet, reported one in two patients reported a significant improvement in abdominal symptoms, abdominal pain, bloating, wind and diarrhea (*p* < 0.02 for all symptoms) [32]. Second, a case-note review of electronic medical records of 88 IBD patients with functional gut symptoms who received low-FODMAP diet advice found a significant reduction in symptoms of any severity (mild, moderate, or severe) for abdominal pain (*p* < 0.001), bloating (*p* < 0.001), flatulence (*p* = 0.041), belching (*p* = 0.001), incomplete evacuation (*p* = 0.012), nausea (*p* = 0.011), and heartburn (*p* =0.035) [33]. Improvements in stool consistency and frequency were observed, including an increase in “normal” stool form (*p* = 0.002) and “normal” stool frequency (*p* < 0.001) [33]. A retrospective, cross-sectional study (*n* = 49) was conducted to investigate long-term adherence and effect on disease course in IBD patients treated with the low-FODMAP diet [34]. Forty-three percent of the IBD patients reported full efficacy (*p* = 0.08) with greatest improvements seen in abdominal pain (63%) and bloating (83%) while on the low-FODMAP diet. The proportion of patients having normal stools increased by 66% in the IBD group (*p <* 0.001). Twenty-four percent of IBD patients became asymptomatic while following the diet. In summary, while there have been no prospective intervention trials completed in IBD, retrospective data suggests that a low FODMAP diet may be a strategy to manage concurrent functional gut symptoms in this population.

### 4.4. Exclusion Diets

Enteral feeding is often used as an adjunctive therapy for maintenance of remission in CD, particularly in Japan. In a randomized control trial, subjects were provided half of the nutrition requirements by an elemental formula (taken orally (*n* = 21) or by nasogastric tube (*n* = 5)), with the remaining 50% of the nutrition requirements met by consuming an unrestricted diet [101]. The relapse rates in the half elemental diet group were significantly lower (34.6% vs. 64.0%; multivariate hazard ratio 0.40 (95% CI: 0.16–0.98)) than the control group after a mean follow-up of 11.9 months. A follow-up report on this study stated that the elemental diet as a maintenance therapy for CD contributed to keeping subjects in a clinically stable state, without affecting their quality of life, nor leading to additional medical expenses [102].

Exclusion diets can be unpalatable and difficult to follow, therefore in a double blind, randomized control trial, the efficacy of a IgG4-guided exclusion diet in subjects with CD was evaluated [44]. The objective of the study was to identify which components of the diet are most important to avoid due to the induction of IgG4, an antibody produced in response to chronic exposure to an antigenic stimulus like a food antigen. It is hypothesized that dietary protein antigens might perpetuate inflammation in CD as a result of previous sensitization. IgG4 titers were tested against 16 common food types. Subjects in the treatment group removed the four food types with the highest antibody titer for four weeks, whereas controls removed the four food types with the lowest antibody titer. The researchers found significant improvements in quality of life, measured by the short inflammatory bowel disease questionnaire (3.05 (0.01–6.11) *p* < 0.05) and disease activity scores, measured by Crohns Disease Activity Index (41 (10.4–71.5) *p* = 0.009). Forty-one percent of subjects receiving the treatment experienced an improvement in Crohns Disease Activity Index score of >100, whereas 16% of controls experienced an improvement. The exclusion of milk, pork, beef and egg was most strongly associated with improvement. This study was underpowered, however, it did demonstrate clinical improvement and this novel approach to dietary management of IBD does warrant further investigation.

### 4.5. Novel Anti-Inflammatory Diet Therapies

While the exact etiology of IBD remains unclear, increasing evidence suggests that the gastrointestinal microbiome plays a critical role in disease pathogenesis [103,104]. Manipulation of the gastrointestinal microbiome through diet interventions, in attempt to reduce systemic inflammation, is increasingly recommended as adjuncts to ongoing medical therapy. The Anti-Inflammatory Diet (IBD-AID) is a nutritional approach designed to address nutrient adequacy, malabsorption and symptoms [45]. IBD-AID restricts the intake of particular carbohydrates (lactose, refined and processed complex carbohydrates), includes the ingestion of pre- and probiotic foods, and modifies dietary fatty acid intake specifically decreasing the total fat, saturated fats, the elimination of hydrogenated oils, and encouraging the increased intake of foods rich in *n*-3 PUFA. A retrospective case series using IBD-AID found that subjects who attempted the diet (*n* = 40), 60% reported reduced symptoms, including reduced stool frequency (patient self-report). A small subset of the subjects (*n* = 7) was able to discontinue at least one of their IBD medications. An intervention trial with a rigorous study design that examines both biomarkers of inflammation, as well as histological changes is needed to further assess efficacy.

### 4.6. Fiber Supplements

The benefits of fiber in IBD are more commonly seen for the use of supplements rather than diet interventions and for the management of UC and CD [105]. Although the evidence is still not clear, interventions with fiber have the potential to relieve symptoms and/or to maintain disease remission in IBD patients. The type of fiber may be an important consideration.

#### 4.6.1. Oat Bran

A controlled intervention study adding 60 grams/day of oat bran (equivalent to 20 grams oat fiber/day) to the diet of subjects (*n* = 22) with quiescent UC reported no signs or symptoms of colitis relapse after 12 weeks [57]. A subgroup of subjects noted a decrease in abdominal pain, reflux and diarrhea (*p* < 0.05). The greatest impact of the oat bran intervention was seen on the fecal short chain fatty acid (SCFA) concentrations found in the stool. Fifteen subjects demonstrated a 36% increase in fecal butyrate concentrations within four weeks (*p* < 0.01) of intervention which was maintained throughout the 12-week intervention. This finding is important as increasing evidence suggests SCFAs play an essential role in maintaining the health of colonic mucosa as butyrate is the main energy substrate for colonocytes [106]. Butyrate also plays an important role in the prevention and treatment of distal UC [107]. Supplementation with oat bran in this population warrants further investigation with a larger sample size, over a longer-term to determine the overall benefit as a maintenance therapy for UC.

#### 4.6.2. Wheat Bran

A randomized control trial completed in CD (*n* = 7) where subjects were instructed to consume a high fiber diet, including consumption of whole wheat bran cereal (1/2 cup daily) and restrict refined carbohydrates, reported improved health-related quality of life as measured by the Inflammatory Bowel Disease Questionnaire (*p* = 0.028) [61]. Significant improvements were seen in the clinical disease activity scores, as measured by partial Harvey-Bradshaw index (*p* = 0.008). This was a feasibility study with a very small sample size (*n* = 4 receiving intervention) that did not include subjects receiving biologic therapies. A much larger sample size, over a longer term, including subjects receiving a variety of medications, is needed to verify the results of this study.

#### 4.6.3. Psyllium

A randomized control trial in subjects with UC in remission (*n* = 105) comparing psyllium fiber (10 g bid) versus mesalamine (500 mg tid) versus psyllium fiber plus mesalamine (10 g bid + 500 mg tid) reported continued remission at 12 months and slightly lower relapse rates in the mesalamine plus psyllium fiber group [52]. Probability of continued remission was similar between all three groups (Mantel-Cox test, *p* = 0.67; intent-to-treat analysis). Hallert et al. (1991) conducted a randomized control trial with UC in remission over four months (*n* = 29) found psyllium (7 g/day testa ispaghula) to be superior to placebo in relieving gastrointestinal symptoms (*p* < 0.001), especially for diarrhea and constipation [53]. Although the dosage of psyllium needs to be elucidated, weak evidence suggests that psyllium fiber may be efficacious in maintaining remission in UC.

#### 4.6.4. Germinated Barley Foodstuff

Germinated barley foodstuff is an insoluble dietary fiber made by milling and sieving brewer’s spent grain [108]. Germinated barley foodstuff has prebiotic properties, containing glutamine-rich protein and hemicellulose-rich fiber which has been shown to reduce clinical activity and prolong remission in UC [58,59,60]. An open-label trial with 41 patients (21 controls, 20 germinated barley foodstuff intervention) with UC in remission received 30 grams (three times daily) of germinated barley foodstuff in addition to conventional medication for two months [59]. A statistically significant reduction in mean CRP was seen in the germinated barley foodstuff intervention group (*p* = 0.017), as well as a significant reduction in abdominal pain and cramping (*p* = 0.017). A similar designed study found that 20 grams of germinated barley foodstuff (*n* = 41) reduced levels of TNF-*α*, IL-6 and IL-8, with significant reductions in IL-6 (*p* = 0.034) and IL-8 (*p* = 0.013) [60]. Length of remission has also been prolonged with long-term administration (12 months) of 20 grams of germinated barley foodstuff [58]. Germinated barley foodstuff appears to be a safe and effective maintenance therapy to prolong remission in patients with UC.

### 4.7. Role of Fat in the Diet

Systematic reviews of the efficacy of *n*-3 PUFA supplementation in maintaining remission in IBD have shown no clear evidence of their efficacy [84,85,109]. However, it may be the type of fatty acids consumed in the diet that are important in maintaining remission. Uchiyama et al. implemented a diet therapy in IBD subjects (*n* = 230) that involved the use of an “*n*-3 PUFA food exchange table” (*n*-3 PUFA diet plan)) to achieve a dietary *n*-3/*n*-6 ratio of [almost equal to]1 [88]. In this regimen, to achieve an *n*-3/*n*-6 ratio of ~1, the *n*-6 PUFA intake was restricted to 50% of the mean intake, and the *n*-3 PUFA intake was increased. The subjects were prohibited from consuming the main sources of dietary *n*-6 PUFA, i.e., vegetable oil; seasonings such as margarine, dressings, and mayonnaise; food cooked in vegetable oil; and snacks. In a subset of subjects, the mean *n*-3/*n*-6 ratio significantly increased after intervention. The mean *n*-3/*n*-6 ratios in the remission were significantly higher than relapse groups (0.65 ± 0.28 and 0.53 ± 0.18, *p* < 0.001) and increase in the *n*-3/*n*-6 ratio was seen in the erythrocyte membrane of IBD subjects. This study concluded that *n*-3/*n*-6 ratio may influence disease activity in IBD subjects. 

The Mediterranean diet pattern may have a protective effect on IBD, as the incidence of IBD in the south of Europe is lower than in northern Europe [110]. The Mediterranean diet pattern is a diet that is high in fiber-rich plant-based foods (e.g., cereals, fruits, vegetables, legumes, nuts, seeds and olives), with olive oil as the principle source of added fat, along with high to moderate intakes of fish and seafood, moderate consumption of eggs, poultry, dairy products (cheese and yogurt), wine and low consumption of red meat [111]. A growing body of scientific evidence indicates that the Mediterranean diet pattern has been associated with significant improvements in health status and [77,78] decreases in inflammatory markers [79]. The protective effect is hypothesized to be derived from the balance in the omega-6/omega-3 ratio of the Mediterranean diet pattern (35% total fat: 15% MUFA (mainly from olive oil), 13% SFA, and 6% PUFA [80]. The mechanisms of how MUFA might be beneficial in colitis are unknown, although adherence to the Mediterranean diet pattern has been shown to beneficially affect the gut microbiome and gut metabolites (metabolome) [35]. A recent case-control study (*n* = 264 IBD subjects and 203 controls) found that low adherence to the Mediterranean diet pattern was a significant risk factor in the development of pediatric UC (OR: 2.3; 1.2–4.5) [36]. An intervention study examining the impact of the Mediterranean diet pattern in CD (*n* = 8) demonstrated a trend for reduction in inflammatory biomarkers (*p* = 0.39) and a tendency for “normalization” of the gut microbiota [37]. The challenge of this study was the lack of statistically significant results and the small sample size. Although a clearer understanding of the role of the Mediterranean Diet Pattern and its impact on IBD is needed, the Mediterranean Diet Pattern may offer a promising approach to reducing markers of inflammation and normalizing the microbiota, but this will need to be confirmed in future clinical trials.

### 4.8. Popular Diet Plans with Patients

The Specific Carbohydrate Diet™ is one of the most popular diets in the lay literature used by patients with IBD. Unfortunately, there is a lack of evidence-based published data on this diet. To date, the evidence for this diet is based on retrospective surveys and case reports. Based on the book “Breaking the Viscous Cycle”, the diet is a strict grain free, sugar-free and complex carbohydrate free diet regimen [112]. An Internet survey (*n* = 451) that examined the IBD patient’s perceptions of the Specific Carbohydrate Diet™, reported that symptoms decreased (abdominal pain, diarrhea, and blood in stool) [38]. Forty-two percent of patients believed the diet helped them achieve remission. A smaller internet survey (*n* = 51) of patients that had used the Specific Carbohydrate Diet™ for management of IBD reported 84% improved on the diet with 75% reporting improved symptoms [113]. Fifty-four percent reported that they maintained remission through use of the Specific Carbohydrate Diet™. In a case-series report (*n* = 50), thirty-three subjects (66%) noted complete symptom resolution at a mean of 9.9 months (range 1 to 60 months) after starting the Specific Carbohydrate Diet™ [41]. Patients’ self-report of the effectiveness of the Specific Carbohydrate Diet™ was rated as a mean of 91.3% effective in controlling acute flare symptoms (range = 30% to 100%) and a mean of 92.1% effective at maintaining remission (range = 53% to 100%). There are two retrospective studies conducted in pediatric subjects that have examined the outcomes of the Specific Carbohydrate Diet™ on clinical outcomes and laboratory parameters in IBD [39,40]. Twenty six patients with IBD (*n* = 20 with CD, 6 with UC) plus 10 controls were analyzed [39]. A comparative analysis of the subjects on the Specific Carbohydrate Diet™ versus controls, revealed significant improvement in Pediatric Crohn’s Disease Activity Index, CRP, and fecal calprotectin over time for both groups (*p* = 0.03, 0.03 and 0.03, respectively). Successful maintenance of remission with the Specific Carbohydrate Diet™ allowed some subjects to discontinue medications and maintain disease control on the Specific Carbohydrate Diet™ alone. A small retrospective chart review (*n* = 11) where pediatric patients followed the Specific Carbohydrate Diet™ for one year found significant improvements in hematocrit, albumin and ESR (*p* = 0.006, 0.002, 0.002, respectively) [40]. Ten children had improvements in weight percentile and nine children had increases in height percentile while following a strict Specific Carbohydrate Diet™. Rigorous prospective, RCTs in pediatrics and adults are needed to determine the merits of this diet for management of IBD.

The Paleo Diet is another popular diet amongst patients with IBD. It recommends avoidance of processed food, refined sugars, legumes, dairy, grains and cereals, and instead it advocates for grass-fed meat, wild fish, fruit, vegetables, nuts and “healthy” saturated fat [114]. While it makes sense that a diet that promotes avoidance of refined and extra sugars and processed energy dense food would have health effects, there are no clinical trials that have examined the efficacy of this diet for IBD. Randomized controlled studies are required to determine whether the Paleo diet has beneficial effects over other diet advice.

## 5. Pharmaconutrition

### 5.1. Curcumin (Turmeric)

Curcumin is the active ingredient found in turmeric, a common Indian spice. Turmeric is commonly used in Ayurvedic medicine for managing a variety of inflammatory diseases. The yellow pigment (curcumin) present in turmeric has been shown to exhibit numerous anti-inflammatory properties [115]. Curcumin is mainly administered orally in the form of capsules filled with its powder. A randomized controlled trial of 82 subjects with UC demonstrated that curcumin at a dose of 2 g/day (1 g/day following breakfast and 1 g/day following supper), when added to standard therapy, significantly reduced relapse rates [54]. Two subjects relapsed during 6 months of therapy with concurrent curcumin therapy (4.65%), whereas 8 of 39 subjects (20.51%) in the placebo group relapsed (*p* = 0.040). Clinical Activity Index and Endoscopic Activity Indices in the curcumin group significantly improved at six months in subjects with UC in remission (Clinical Activity Index: *p* = 0.038 and Endoscopic Activity Indices: *p* = 0.0001). A systematic review [55] and one Cochrane review [56] conclude that curcumin may be an effective and safe therapy for maintaining remission in UC for up to six months when provided with standard therapy (sulfasalazine and mesalamine). More extensive, well-controlled clinical trials are needed to confirm the benefits of curcumin, at present it appears to be a safe, low cost option for maintenance of remission in UC when added to standard medical therapy.

### 5.2. Probiotics

The use of probiotics in the maintenance of remission in IBD has been investigated in several clinical trials, however results are inconsistent. The clinical studies have significant heterogeneity, including a variety of different genera, species, strains and doses of probiotics that have been examined, which makes it difficult to draw firm conclusions about efficacy. A meta-analysis of the probiotic strain *Escherichia coli* Nissle 1917 in maintenance of remission in UC found that *E.coli* Nissle 1917 maintained remission as well as standard therapy with mesalazine (OR = 1.07, 95% CI: 0.70–1.64) [116]. Similarly, another meta-analysis showed that probiotics could prevent relapse of UC as effectively as mesalazine (*n* = 638; 316 probiotic group, 322 mesalazine group; RR = 1.0, 95% CI: 0.79–1.26) [117]. The strains shown to be effective were *E. coli* Nissle 1917, *E. coli* (serotype O6:K5:H1), *Lactobacillus* GG and Probio-Tec AB-25 (*L.s acidophilus* La-5 and *Bifidobacterium animalis* subsp. lactis BB-1). Shen et al., (2013) also found that probiotics were comparable to 5-ASA in maintaining therapy in UC (*p* = 0.69, RR = 0.96) [118]. In contrast, a Cochrane collaboration found no difference between probiotics and mesalazine for maintenance of remission in UC (3 studies; 555 patients: OR 1.33; 95% CI: 0.94–1.90) [119]. Supplementation with probiotics in the maintenance of remission in CD is also unfavorable. Two meta-analyses found that the probiotic strains *Lactobacillus* GG and *Lactobacillus johnsonii* LA1 could not prevent relapse in CD (RR = 1.18, 95% CI: 0.81–1.70) [118,120] and concluded that probiotics had no significant benefit in CD with probiotic supplementation (*p* = 0.71, RR = 1.09) [120]. The probiotic strain *Lactobacillus* GG was shown to have significantly benefit in favor of relapse versus the placebo (RR 1.68, 95% CI: 1.07 to 2.64) [118]. Future studies examining probiotics in the maintenance of remission in IBD need to focus on the effects of different probiotic strains and different dosages, together with the homogeneous patient populations to determine which patients are most likely to benefit from probiotic treatment.

### 5.3. Vitamin D

Vitamin D deficiency is common in IBD patients with a frequency ranging from 16% to 95% [121]. One study reports that patients with UC had more than double the odds of vitamin D deficiency when compared with normal controls (OR = 2.28; 95% CI: 1.18–4.41; I = 41%; *p* = 0.01) [47]. There is also evidence to suggest that vitamin D deficiency may influence the severity of inflammation in IBD [48,49,50]. A cross-sectional study revealed that subjects with active disease had more frequent low vitamin D levels (80% vs. 50.4%, *p* = 0.005) when compared to subjects in remission [49]. A large prospectively collected cohort of 230 subjects demonstrated an inverse association between serum 25(OH)D concentrations and mucosal inflammation, as assessed by the Mayo endoscopy score (*p* = 0.01), disease activity as indicated by the total Mayo score (*p* = 0.001), and histologic activity (*p* = 0.02) in subjects with UC [48]. Serum 25(OH)D concentrations have been found to be inversely correlated with fecal calprotectin (*r* = −0.207, *p* = 0.030), particularly among CD subjects in clinical remission (*r* = −0.242, *p* = 0.022) [50]. Furthermore, a large cohort of over 3200 IBD patients demonstrated that low plasma 25(OH)D level (<50 nmol/L) was independently associated with an increased risk of subsequent surgery (OR: 1.76; 95% CI: 1.24–2.51) and hospitalization (OR: 2.07; 95% CI: 1.59–2.68) in CD with similar results found in UC [51]. A study that followed IBD patients for five years (*n* = 965) found that patients with low mean vitamin D levels (<50 nmol/L) had worse pain, disease activity scores, health care utilization and quality of life (*p* < 0.05) compared with subjects with normal mean vitamin D levels (>75 nmol/mL) [122]. A large-multi-institutional cohort of IBD patients found that patients with vitamin D deficiency (<50 nmol/L) had an increased risk of cancer (OR: 1.82; 95% CI: 1.25–2.65) compared to those patients that had sufficient levels [123].

Given that vitamin D status appears to be an independent risk factor for potential poorer outcomes in IBD, supplementation of vitamin D in this population is judicious. Patients with CD who were deficient in vitamin D (<75 nmol/L) and normalized their 25(OH)D had a reduced likelihood of surgery (OR: 0.56; 95% CI: 0.32–0.98) compared with those who remained deficient [51]. A prospective study in UC (*n* = 70) demonstrated that low vitamin D levels (<88 nmol/L) increased the risk of clinical relapse over 12 months, as well as was associated with increased presence of endoscopic inflammation (OR: 1.29; 95% CI: 1.07–1.85; *p* < 0.01) or histologic inflammation (OR: 1.46; 95% CI: 1.13–1.88; *p* = 0.005) [124]. The optimal concentration for patients with IBD remains unknown, but targeting serum 25(OH)D concentrations above 75 nmol/L appears safe and may have benefits for IBD disease activity, improving bone health, preventing colo-rectal cancer, and alleviating depression [125]. Daily doses of 1800–10,000 IU of vitamin D3 (cholecalciferol) are probably necessary, depending on the baseline vitamin D serum concentration, ileal involvement in CD and body mass index [125]. In a clinical trial by Jorgensen et al. (2010) (*n* = 108), in CD, oral vitamin D3 treatment with 1200 IU daily increased serum 25(OH)D from mean 69 nmol/L to mean 96 nmol/L after three months of treatment (*p* < 0.001) [126].

## 6. Challenges in Creating Evidence-Based Guidelines

Although there are diet intervention trials that show promise in maintaining remission, their efficacy remains in question. The short duration of the interventions (less than 12 weeks), the lack of a proper control group in some instances, and the small sample sizes (less than 20 individuals) make it very challenging for clinicians to draw firm conclusions from existing data. There is an overall lack of objective clinical and endoscopic disease markers. For example, many studies are completed retrospectively and they rely on patient questionnaires regarding disease symptoms, such as pain and stool frequency. Well-designed clinical trials in IBD are urgently required to define the precise role of each of these diets in the prevention or management of IBD. Up until now, the role of diet in IBD is highly undermined by lay and anecdotal reports without sufficient scientific proof. 

High quality diet intervention studies for the treatment of IBD need to include the following: (1) quantification of baseline intake of the habitual diet; (2) monitoring of diet adherence through food recalls; (3) large prospective, control trials over a longer-term; (4) use of a control diet to determine the specificity of observed effects to the intervention; (5) use of a variety of endpoints (symptoms, quality of life, clinical biomarkers, endoscopic indices, diet and fecal assessment) to monitor response to diet interventions; and (6) consider the use of IBD animal models (e.g., gnotobiotic mouse model) to discover the mechanisms of pathogenesis.

As research in this field moves forward, a personalized approach to nutrition for individuals or for subsets of patients may be the next frontier. A personalized approach may not only be useful for primary prevention of IBD but also treating disease. We must be mindful that a generalized diet treatment for all patients may not work. Certainly, we are seeing emerging research to suggest that microbiota-targeted approaches have demonstrated some promise for managing chronic disease [127].

## 7. Conclusions

As our knowledge of the associations between a disrupted intestinal microbiota (dysbiosis) and chronic inflammatory diseases expands, the influence of diet becomes increasingly important. Clearly, diet and nutrition are of major interest for patients with IBD. Patients use a variety of diet strategies in attempt to manage underlying disease, as well as to provide relief from symptoms. Diet plays a key role in IBD pathogenesis, and there is a growing appreciation that the interaction between diet and microbes in a susceptible person contributes significantly to the onset of disease [128]. Several lines of evidence point to aspects of the typical Western diet that may promote the development of IBD. A low-residue diet is frequently recommended for IBD by health professionals [19] to reduce symptoms may be adding further insult. A diet that lacks dietary fiber may accelerate dysbiosis in IBD [94,95].

Pre-illness studies in IBD and intervention trials provide convincing evidence that a plant-based diet, with increased consumption of fruit/vegetables and less red meat intake could be suggested to patients with IBD in remission. Several of the diets/supplements discussed in this review appear to hold promise for in the maintenance of remission in IBD, especially when provided in addition to standard medical therapy.

## Figures and Tables

**Table 1:** Evidence-based diet recommendations for the maintenance of remission in IBD.

Statement	Recommendation
Encourage high dietary fiber intake from foods, especially from fruits and vegetables [22,23,24,25]	Strongly Recommend *
Avoid *n*-6 PUFA (safflower oils, corn oils, margarine) and trans-unsaturated fatty acid consumption [23,24,26,27]	Strongly Recommend
Encourage consumption of dairy products [28,29]	Recommend † (if tolerated)
Limit/avoid refined carbohydrates, especially sweetened beverages and soft drinks [30]	Recommend
Limit red meat consumption, especially from beef, pork, lamb and processed meats [22,31]	Recommend
FODMAP Diet [32,33,34]	Optional ‡ (for the management of IBS-overlay)
Mediterranean Diet Pattern [35,36,37]	Optional
Specific Carbohydrate Diet [38,39,40,41]	No Recommendation ^β^
Low Residue Diet [42]	
Semi-vegetarian Diet [43]	
IgG4-guided Elimination Diet [44]	
IBD-AID [45]	
Paleo Diet	No Recommendation

Evidence graded according to the American Academy of Pediatrics, Steering Committee on Quality Improvement and Management—“Classifying recommendations for clinical practice guidelines” [46]. * Strongly Recommend: The quality of the supporting evidence is excellent, based on well-designed randomized control trials (RCTs) and/or consistent evidence from observational studies. Benefit clearly outweighs harm. † Recommend: The quality of the supporting evidence is good, but RCTs and/or evidence from case-control/cohort studies has limitations. Anticipated benefits outweigh harm. ‡ Optional: The quality of evidence is suspect, further well-performed studies needed. May be of limited advantage, however there is still unclear balance between benefit and harm. ^β^ No recommendation: There is a lack of or poor evidence. Unclear balance between benefit and harm.

**Table 2:** Evidence-based nutrition recommendations for supplements in the maintenance of remission in IBD.

Statement	Type of IBD	Recommendation
Vitamin D (minimum 1200 IU/day) [47,48,49,50,51]	Both	Strongly Recommend * (aim for levels of serum 25 (OHD) >75 nmol/L
Psyllium (minimum 4 grams/day) [52,53]	UC	Recommend †
Curcumin (1-gram bid) [54,55,56]	UC	Optional ‡
Oat bran supplementation (20 grams/day) [57]	UC	Optional
Germinated Barley Foodstuff (minimum 20 grams/day) [58,59,60]	UC	Optional
Wheat bran (1/2 cup daily) [61]	CD	Optional

Evidence graded according to the American Academy of Pediatrics, Steering Committee on Quality Improvement and Management—“Classifying recommendations for clinical practice guidelines” [46]. * Strongly Recommend: The quality of the supporting evidence is excellent, based on well-designed randomized control trials (RCTs) and/or consistent evidence from observational studies. Benefit clearly outweighs harm. † Recommend: The quality of the supporting evidence is good, but RCTs and/or evidence from case-control/cohort studies has limitations. Anticipated benefits outweigh harm. ‡ Optional: The quality of evidence is suspect, further well-performed studies needed. May be of limited advantage, however there is still unclear balance between benefit and harm. ^β^ No recommendation: There is a lack of or poor evidence. Unclear balance between benefit and harm.
